# PTSD and Depression in Healthcare Workers in the Italian Epicenter of the COVID-19 Outbreak

**DOI:** 10.2174/1745017902117010242

**Published:** 2021-12-24

**Authors:** Claudia Carmassi, Virginia Pedrinelli, Valerio Dell’Oste, Carlo Antonio Bertelloni, Chiara Grossi, Camilla Gesi, Giancarlo Cerveri, Liliana Dell’Osso

**Affiliations:** 1 Department of Clinical and Experimental Medicine, University of Pisa, Pisa, Italy; 2 Department of Biotechnology, Chemistry and Pharmacy, University of Siena, Siena, Italy; 3 Department of Mental Health and Addiction, ASST Lodi, Lodi, Italy; 4 Department of Mental Health and Addiction, ASST Fatebenefratelli Sacco, Milan, Italy

**Keywords:** Healthcare workers, Stress, Post-Traumatic Stress Disorder, Depression, Psychological distress, COVID-19

## Abstract

**Background:**

Increasing evidence highlights the susceptibility of Healthcare Workers to develop psychopathological sequelae, including Post-Traumatic Stress Disorder (PTSD) and depression, in the current COronaVIrus Disease-19 (COVID-19) pandemic, but little data have been reported in the acute phase of the pandemic.

**Objective:**

To explore Healthcare Workers’ mental health reactions in the acute phase of the COVID-19 pandemic in the first European epicenter (Lodi/Codogno, Italy), with particular attention to post-traumatic stress and depressive symptoms and their interplay with other psychological outcomes.

**Methods:**

74 Healthcare Workers employed at the Azienda Socio Sanitaria Territoriale of Lodi (Lombardy, Italy) were recruited and assessed by means of the *Impact of Event Scale- Revised*, the *Professional Quality of Life Scale-5*, the *Patient Health Questionnaire-9*, the *Generalized Anxiety Disorder-7 item*, the *Resilience Scale* and the *Work and Social Adjustment Scale*. Socio-demographic and clinical variables were compared across three subgroups of the sample (No PTSD, PTSD only, PTSD and depression).

**Results:**

A total of 31% of subjects endorsed a diagnosis of PTSD and 28.4% reported PTSD comorbid with major depression. Females were more prone to develop post-traumatic stress and depressive symptoms. Subjects with PTSD and depression groups showed high levels of PTSD, depression, burnout and impairment in functioning. Anxiety symptoms were higher in both PTSD and depression and PTSD groups rather than in the No PTSD group.

**Conclusion:**

Our results showed high rates of PTSD and depression among Healthcare Workers and their comorbidity overall being associated with worse outcomes. Current findings suggest that interventions to prevent and treat psychological implications among Healthcare Workers facing infectious outbreaks are needed.

## 1 INTRODUCTION

In Europe, the first case of pneumonia diagnosed as COronaVIrus Disease-19 (COVID-19) was recorded on February 20^th^, 2020 at the public hospital of Codogno (Lodi), in the north Italian region of Lombardy. On February 22^nd^, 76 additional cases were confirmed, so the epidemic had begun and Italy was considered the European epicenter of the emergency. Since the beginning of the COVID-19 health emergency, Healthcare Workers (HCWs) have been on the frontline fighting the emergency with a high toll, providing care despite exhaustion, personal risk for infection, fear of transmission to family members, illness or death of friends and colleagues and the loss of many patients [1, 2].

If in the beginning, in fact, many people minimized the problem and believed that we were dealing with something “just like the flu”, healthcare personnel facing the outbreak of the pandemic in northern regions of Italy first warned that the risk was absolutely underestimated. Despite most hospitals had plans in place for a COVID-19 outbreak upon the first Chinese reported evidence, few, if any, had imagined that it would have been so rapidly spreading and devastating. For these reasons, HCWs facing the first phase of the emergency were quite unprepared and they abruptly fell in a “war-like” setting [3, 4].

The susceptibility of HCWs to develop psychological distress related to their work activity, particularly of those involved in frontline activity, is well documented by literature published in the last decades. Repeated exposure to stressful events, especially in the context of emergency care settings, can exert a deep influence on the mental state and global functioning of HCWs, up to the point of leading to the onset of a wide range of psychopathological sequelae, such as Post-Traumatic Stress Disorder (PTSD), depression, burnout and anxiety [5-8]. However, little data are available about the psychological impact of the COVID-19 emergency in the acute phase in Italy among HCWs [9-12], in spite of the immediate attention drawn on the mental health of HCWs’ facing this new challenge [13].

Data from previous viral outbreaks highlighted considerable rates of psychological comorbidities among HCWs, such as distress, depression, anxiety and burnout, especially in those providing frontline healthcare activity, with both short and long-term consequences [14-17]. Not unexpectedly, studies on samples of HCWs from different countries facing the first phase of the COVID-19 pandemic close to the peak of contagion also found remarkable rates of post-traumatic stress reactions (ranging from 37% to 71%), depression (besides 24% to 50%), anxiety (going from 19% to 44%) and other unfavourable mental health outcomes with a negative impact on work and social adjustment [18-23]. Many risk factors of sociodemographic and occupational nature were shown to be associated with the likelihood of developing mental health problems in HCWs during and after viral outbreaks, among which were female gender, younger age, working in a high-risk environment (such as the frontline activity), higher perception of threat and risk, lack of specialised training received and isolation [24-26].

The COVID-19 emergency soon showed to embody potentially stressful characteristics in their extreme manifestations, as HCWs were facing an unprecedented toll of critical patients who showed a specific clinical picture characterized by severe distress, worsening dyspnoea and high mortality rates [26]. The extreme rapidity of the contagion propagation and the massive load of patients needing hospital care and ICU intervention constituted two of the main characteristics of this sanitary burden besides the frequent need for patients’ management in stringent isolation [27].

Further issues contributed to making this scenario even more challenging. On the one hand, especially in the most affected areas of Northern Italy, a condition of scarcity of resources occurred, forcing health professionals to deal with an extreme decision-making burden, thus making the sense of helplessness pervasive [28, 29]. On the other, HCWs assisted patients facing the most critical moment of their lives in total isolation, and so they represented the only means of communication with the outside world and especially with family members [30].

The need to define effective strategies to preserve both physical and mental health of HCWs exposed to the current health emergency became urgent soon: WHO called for immediate action [26] and some intervention strategies were proposed [1, 31]. In this framework, clinicians of the Psychiatric Unit of the Codogno and Lodi Hospitals rapidly put in place strategies to provide mental health support to first-line HCWs and promptly started a collaboration with researchers of the Psychiatric Clinic of the University of Pisa, while delineating the first supportive measures to Healthcare personnel facing the ongoing COVID-19 public health emergency [1, 9].

Despite a large amount of data available on the mental toll for HCWs driven by the still ongoing COVID-19 pandemic, little data are on the interplay role of the different psychopathological outcomes explored to gain a complete overview.

On these premises, the aim of the present study was to explore mental health in a sample of HCWs facing the acute phase of the COVID-19 pandemic in the Italian epicenter seeking psychological support, with particular attention to PTSD, depression and their comorbidity. Furthermore, we examined the interplay between these two disorders and psychiatric symptoms, functioning levels and professional quality of life.

## 2 METHODS

### 2.1 Study Sample and Procedures

The study sample included a consecutive sample of 74 HCWs employed at the Azienda Socio Sanitaria Territoriale (ASST) of Lodi (LO, Lombardy, Italy) during the initial phase of COVID-19 pandemic. Subjects were consecutively enrolled in the psychiatric outpatients’ service of the Ospedale Civico of Codogno (LO, Lombardy, Italy) and the Ospedale Maggiore of Lodi (LO, Lombardy, Italy), dedicated to HCWs, between 1^st^ April, 2020 and 31^st^ May, 2020. The service was specifically introduced to provide a prompt response to the psychological pressures, which the hospital staff was facing within the context of the COVID-19 emergency. In this framework, clinicians and researchers of the Psychiatric Clinic of the University of Pisa (Italy) started collaborating with the Psychiatric Units of Codogno and Lodi Hospitals, in order to define adequate measures to assess and prevent adverse mental health outcomes [8]. According to the study protocol, HCWs facing the COVID-19 Emergency who approached the service voluntarily seeking psychological support were asked to participate in the study. Exclusion criteria included poor knowledge of the Italian language or other limits to verbal communication. Suitable subjects were asked to provide written informed consent after receiving a complete description of the study, having the opportunity to ask questions. The study was conducted in accordance with the Declaration of Helsinki and approved by the local Ethics Committee (Area Vasta Nord Ovest, Protocol number 17151/2020).

### 2.2 Instruments and Assessments

The whole sample was investigated by means of psychometric instruments, including the *Impact of Event Scale- Revised* (IES-R) [32], to investigate post-traumatic stress symptoms; the *Patient Health Questionnaire-9* (PHQ-9) [33] to examine depressive symptoms; the *Professional Quality of Life Scale-5* (ProQOL-5) [34] to investigate Compassion Satisfaction and Compassion Fatigue related to work activity; the *Generalized Anxiety Disorder 7-Item* (GAD-7) [35] to explore anxiety symptoms; the *Resilience Scale* (RS) [36] to investigate resilience level; the *Work and Social Adjustment Scale* (WSAS) [37] to assess impairment in work and social functioning. Further, socio-demographical data were collected through a specific datasheet reporting information on the COVID-19 pandemic.

The IES-R is a 22-item scale measuring three core phenomena of PTSD, *i.e*., re-experiencing of traumatic events, defensive avoidance and denial of trauma related memories and emotions. It refers to the last week. The questionnaire has an adequate internal consistency (Cronbach’s α = .80 - .93 for the intrusion; Cronbach’s α = .73 - .84 for avoidance), and high test-retest reliability (r = .93). A score between 24 and 32, indicates a partial PTSD diagnosis, whereas a score over 32 represents a cutoff for PTSD diagnosis [32]. According to the aim of the study, the items referred to the traumatic events that the subjects had experienced in the framework of their working activity in the hospital.

The PHQ-9 represents one of the most used self-assessment tools for the screening of depressive symptoms. It consists of 9 items that investigate the presence of depressive symptoms in the last two weeks, each evaluated on a 4-point scale from 0 (never) to 3 (almost every day) [30]. An overall score greater than or equal to 10 indicates the presence of depression. The test has adequate internal consistency (Cronbach’s α= .89) and excellent test-retest reliability (r = .84).

The ProQOL-5 [34] is a 30- item self-report measure assessing two aspects: *Compassion Satisfaction* (CS) and *Compassion Fatigue* (CF) related to work. CF integrates two factors, namely *Burnout* and *Secondary Traumatization* (ST), related to the work-related secondary exposure to stressful events. Respondents were asked to indicate how often (from 1-never to 5-very often), during the last 30 days, each item was experienced. In the CS dimension, which measures the positive aspects derived from being able to do work well, high scores represent a greater satisfaction related to the ability to be an effective caregiver. The *Burnout* dimension in this scale is associated with feelings of hopelessness and difficulties in dealing with work. The ST dimension is related to the work-related secondary exposure to stressful events. High scores indicate exposition to frightening experiences at work. The category raw scores may range from 10 to 50; score ranges are also available for each category. Internal consistency was good for the CS scale (Cronbach's α = .88) and adequate for the Burnout and ST scales (Cronbach's α = .75 and = .81, respectively) [31].

The GAD-7 is a self-assessment questionnaire used as a tool for screening and measuring the severity of anxious symptoms. Particularly, it investigates the frequency of anxious symptoms in the last two weeks using 7-item with a score ranging from 0 (never) to 3 (almost every day) [35]. Scores over 10 suggest the presence of Generalized Anxiety Disorder (GAD). The internal consistency of the GAD-7 is excellent (Cronbach’s α = .92) and test-retest reliability is also good (r= .83).

The RS was developed in order to investigate the degree of individual resilience, considered a positive personality characteristic that enhances individual adaptation. The Italian version of the RS is a 24-item Likert scale using a 7- point rating (1 disagree - 7 agree); the score ranges from 24 to 164 [36]. The Italian version of the questionnaire has a good internal consistency (Cronbach's α = .86).

The WSAS is a test used to evaluate and measure work and social adjustment. It includes five items that assess the individual’s ability to perform the activities of everyday life and how these are affected in the week prior to the assessment [37]. The first item investigates the work ability of the subject; the second item assesses the ability to cope with household chores; the third item assesses private recreational activities; the fourth and fifth items investigate the family interaction and relationship, respectively. Each of the five items is rated on a nine-point scale ranging from 0 (not at all) to 8 (severe interference); hence the total scores range from 0 to 40. The internal consistency (Cronbach’s α) of the instrument varies from .70 to .94 and the reliability of the test-retest is 0.73.

### 2.3 Statistical Analysis

All statistical analyses were performed using the Statistical Package for Social Science, version 25.0 (SPSS Inc.). Continuous variables were reported as mean ± standard deviation (SD), whereas categorical variables were reported as percentages. All tests were two-tailed and a p value <.05 was considered statistically significant.

Statistical analysis included descriptive and comparative analysis. Descriptive procedures were used to evaluate frequencies of PTSD and depression in the sample.

Through comparative analysis, the three subgroups of the sample (No PTSD, PTSD only, PTSD and depression) were compared according to socio-demographic, work-related and clinical variables. Group comparisons were performed by using the Chi-square likelihood ratio for categorical variables (including socio-demographic and work-related ones) and the non-parametric Kruskal-Wallis test, followed by Dunn test for pairwise comparisons for continuous variables (socio-demographic features, IES-R, PHQ-9, ProQOL-5, GAD-7, RS and WSAS scores).

## 3 RESULTS

The study included a total sample of 74 HCWs, 27 (36.5%) males and 47 females (63.5%). The mean age in the total sample was 39.3±12.2 years. Regarding employment status, 18 (24.3%) participants were medical doctors, whilst 56 (75.7%) were other HCWs (nurses, administrative staff) and 18 (24.3%) worked in the Emergency Rescue (ER) unit. Regarding COVID-19 emergency, 46 (62.2%) HCWs were directly involved in the management of COVID-19 patients, 39 (52.7%) reported having assisted at least one patient who died because of the COVID-19 and 45 (60.8%) referred an increased workload linked to the emergency. The death of a relative or a close one due to the COVID-19 was reported by 13 (17.8%) subjects and about half participants (35, 47.3%) had at least one relative or a close one who got sick with COVID-19. Regarding clinical features, 15 (20.3%) had a positive family history of mental disorders, 14 (18.9%) reported having been under psychiatric treatments and 13 (17.6%) were suffering from some physical illnesses. Socio-demographics and work-related features of the study sample were reported in Table **1**.

The IES-R mean total score was 38.4±18.6, whereas the mean scores of the *intrusion*, *avoidance* and *hyperarousal* domains were respectively 1.9±1.0, 1.5±7.7 and 1.5±1.0. According to the IES-R, 23 (31%) HCWs endorsed a diagnosis of probable PTSD (IES-R>33). The PHQ-9 mean score was 7.7±5.2. The scores reported by any of the participants screened positive for only clinical depression (PHQ-9>10), but, interestingly, according to the IES-R and the PHQ-9 scales, 21 (28.4%) subjects reported PTSD comorbid with depression. The three ProQOL subscales (CS, Burnout and ST) mean scores were 37.9±6.4, 23.4±6.0 and 22.4±8.0, respectively. Furthermore, in the total sample, the mean scores of GAD-7, RS and WSAS were 9.7±5.6, 129±21.9 and 16.2±11.1, respectively. According to IES-R and PHQ-9 scores, the sample was divided into three subsamples: subjects without PTSD (No PTSD group), subjects with PTSD only (PTSD only group) and subjects with both PTSD and depression (PTSD and depression group).

Comparison of socio-demographics and COVID-19 related characteristics of the sample among the three groups were shown in Table **1**. Particularly, a gender difference emerged, with females reporting significantly higher rates of PTSD and depression with respect to males (85.7% vs 14.3% respectively, p=.032). Percentages of subjects reporting threshold PTSD and both PTSD and depression according to IES-R and PHQ-9 cut-off scores in the overall sample and divided by gender were reported in Fig. (**1**).

Between group comparisons of continuous variables were performed by using the Kruskal-Wallis test and summarized in Table **2**. Particularly, statistically significant differences among the three groups emerged in the IES-R total (p<.001) and intrusion (p=<.001), avoidance (p<.001) and hyperarousal (p<.001) domains scores, besides in the PHQ-9 (p<.001), ProQOL Burnout (p<.001) and ST (p<.001) subscales, GAD-7 (p<.001) and WSAS (p<.001) scores. Post-hoc comparisons across the three groups revealed that, compared to No PTSD group, subjects with PTSD only and both PTSD and depression reported more severe post-traumatic stress symptoms (PTSS) as measured by the IES-R total and each domain scores. Subjects with both PTSD and depression also showed higher levels of depressive symptoms on PHQ-9 questionnaire as compared to the two other groups. Further, HCWs of the PTSD and depression group experienced significantly higher levels of Burnout and ST with respect to the other HCWs groups; moreover, PTSD group also reported higher levels of ST with respect to No PTSD one. Anxiety symptoms, assessed by GAD-7 scores, were more severe among individuals with both PTSD and depression rather than among those without PTSD, with ones with PTSD showing also higher GAD-7 scores than those without. Finally, impairment in work and social functioning as measured by WSAS scale was significantly higher in the PTSD and depression group as compared to the two others.

## 4 DISCUSSION

The results of the present study highlighted severe PTSD symptoms, compatible with probable PTSD diagnosis, among 31% of the HCWs facing the acute phase of the COVID-19 outbreak (April-May, 2020) in the first Italian epicenter. Such rates resulted substantially higher with respect to those found in other samples of HCWs at the peak of the COVID-19 pandemic [13, 35]. In a study on more than 600 Healthcare professionals working in a tertiary hospital in the first Chinese epicenter in the first phase of the COVID-19 emergency, the prevalence of PTSD was about 20% [38], whilst a survey on over 2500 HCWs in the United Kingdom [21], as an instance, reported rates of PTSD as high as about 24% [19]. Our findings showed higher rates of psychological distress also with respect to other studies exploring the early psychiatric impact of COVID-19 emergency in other clinical samples [20, 39], as well as in HCWs in Italy [11, 12, 40] and in the Lombardy region of the Country [10]. Conversely, our results are in line with a study on almost 700 HCWs investigating post-traumatic stress in the acute phase of the pandemic in Lombardy, with a substantial share of HCWs (38.9%) who received a provisional diagnosis of PTSD [12]. We may argue that such high rates of PTSD in our sample were related to the enrollment method that was part of a support program aimed at mitigating the high psychological burden of HCWs who voluntarily asked for help. This finding may also reflect exposure to particularly severe distress in this sample. Indeed, HCWs enrolled were involved in the first outbreak in the Lombardy region that accounted for 37% of cases and 53% of deaths for COVID-19 in Italy, as of April 15, 2020 [41] and they had to learn, on field, clinical characteristics, risks and need for care of the novel COVID-19 infection.

It is now well established that PTSD is not the only psychiatric condition that may emerge in the aftermath of traumatic events and that comorbidity is the norm rather than exception [42-45], with depression being one of the most frequent conditions occurring concurrently with PTSD [46-48]. It has been proposed that, when comorbid in the aftermath of a traumatic event, PTSD and depression might represent a single construct with common predictive variables [48, 49]. Reports from both epidemiological and clinical samples highlighted that risk for depression following trauma increased only among individuals who developed PTSD with respect to those who did not, concluding that PTSD and depression in trauma victims were influenced by the same vulnerabilities [46-52]. Conversely, other studies reported the onset of depression and PTSD as independent sequelae of traumatic events [53, 54] or temporally associated with causality, with depression having often a later onset than that of PTSD [55, 56]. Noteworthy, in the present study, none of the participants screened positive for clinical depression only, whilst about one third of them (28.4%) were identified as having PTSD comorbid with depression. Lower rates of comorbid PTSD and depression were reported in specific trauma-exposed samples, as in the context of natural disasters [57, 58] or terrorist attacks [59-61]. However, few studies explored the burden of PTSD and depression comorbidity among HCWs. Our results are in line with the study by Palgi *et al*. that assessed PTSD and depression among 127 HCWs one month after the 2006, Lebanon and Israel war began: PTSD and depression comorbidity rates were as high as 22.6% and PTSD symptoms were found to be highly associated with increased risk for depression [62].

We report data from a specific sample of HCWs exposed at the beginning of the COVID-19 outbreak revealing considerable rates of PTSD comorbid with depression. This finding is, on the one hand, in line with the hypothesis that these two disorders are part of a shared vulnerability having the same predictive factors in which depressive symptoms are often integral to PTSD [44, 48] and, on the other, maybe due to the early assessment of HCWs after the onset of the emergency. In fact, there is also evidence to suggest that, in the first few months after trauma, depression may exist as a separate construct, with a pattern of onset later compared to that of PTSD [42, 48, 63, 64].

Comparison of demographics and COVID-19 related work characteristics in the three groups (No PTSD, PTSD only, PTSD and depression) revealed no significant differences, except for gender, with females being more prone to develop post-traumatic stress reactions comorbid with depression with respect to males. The female gender has been proved to be a risk factor for both PTSD and depression, supporting the hypothesis of a shared psychopathological vulnerability between the two disorders [65-72]. Recent reviews about the impact of the current and previous infective outbreaks in HCWs also found generally female professionals to experience higher rates of PTSD and depressive symptoms [23, 24, 73]. Despite this, studies exploring the burden of comorbid PTSD and depression in the aftermath of trauma, such as in the context of 9/11 attack or natural disasters found no gender differences, also in the long-term [74, 75]. On the contrary, a large study on over 2400 adults, including subjects with prior or current affective disorder and healthy controls assessed at three times and followed over five years, revealed high rates of PTSD and comorbid psychiatric disorders, in particular among patients with major depressive disorder (84.4%); comorbidity with depression was associated with female gender, neuroticism and symptom severity [76].

Comparisons in the three groups substantially showed HCWs with both PTSD and depression globally reporting a worse psychological outcome with respect to the other two others. Consistent with the previous work [60, 74, 77], patients reporting PTSD with comorbid depression had greater PTSD symptom severity, as shown by the higher IES-R total and each domain scores with respect to the other two groups.

Further, our finding emphasized a bidirectional relationship between PTSD and depression for what concerns symptoms severity. HCWs reporting PTSD and depression, in fact, showed higher rates of depressive symptoms with respect to those with PTSD only. While the evidence is not universal, many studies demonstrated that depressive symptoms co-occurring with PTSD might influence symptoms’ severity and also PTSD treatment outcomes. For example, Campbell *et al*., in a cross-sectional study on a large sample of depressed military veterans primary care patients, estimated PTSD prevalence and compared demographic/illness characteristics of patients with depression alone to those who screened positive also for PTSD, finding that the presence of PTSD and depression comorbidity was associated with more severe depression, more frequent outpatient Healthcare visit and higher suicidal ideation [77]. Another study on almost 1200 psychiatric inpatients found that compared to depressed patients without PTSD, depressed patients with PTSD experienced greater psychiatric symptom severity and higher levels of depression and hostility at discharge. Depressed patients with comorbid PTSD also had a significantly higher rate of being discharged against medical advice and PTSD comorbidity correlated with higher functioning impairment, suggesting recognition of PTSD comorbidity may improve overall care of these patients [78].

Noteworthy, in the present study, the comorbid PTSD and depression group experienced, on the overall, higher levels of Compassion Satisfaction, Secondary Trauma, anxiety and lower levels of work and social functioning compared to the groups reporting PTSD or neither the two disorders. A positive association between burnout and depressive symptoms was previously proved, and some longitudinal studies specifically described a trajectory from depression to burnout [79, 80]. A recent study on 110 emergency HCWs, subjects reporting a DSM-5 PTSD diagnosis showed significantly higher depressive symptoms with respect to those without and a significant correlation emerged among PTSD, burnout and lifetime mood spectrum symptoms, particularly the depressive ones [17].

In the last decades increasing attention was paid to comorbidity between PTSD and anxiety disorders and many authors found that a considerable proportion of PTSD casualties, widely ranging from 20% to 90%, suffered from co-morbid anxiety [63, 81-83] and up to 65% of them met criteria for a triple-comorbidity, namely having anxiety and depression in addition to PTSD [78, 79]. In the present study, the PTSD and depression group reported more severe anxiety symptoms with respect to No PTSD and PTSD only groups. There is a general consensus that co-occurrence of anxiety symptoms in PTSD is associated with more severe PTSD symptoms, greater PTSD-related disability and functional impairment [84-86]. A recent survey investigating the mental health and quality of life of HCWs during the COVID-19 pandemic found that those presenting post-traumatic stress and depressive symptoms also had significantly higher levels of anxiety and perceived risk for developing serious complications resulting from COVID-19 infection compared to those without [87, 88]. Data from a further survey on HCWs after the peak of the pandemic in China pointed out a PTSD prevalence as high as 20.8%; among HCWs affected by PTSD, over 85% of them reported varying degrees of anxiety and depression and generally had higher levels of depression and anxiety as compared to those without [38].

Several authors demonstrated the additional burden due to comorbidity with depression in PTSD in terms of functional impairment as well as reduced quality of life [49, 53, 74, 75], also among health professionals [6, 8, 84]. Findings from previous outbreaks first highlighted the risk for HCWs experiencing adverse mental health outcomes, included post-traumatic and depressive symptoms, for a negative impact on their global functioning [23, 31] and a few studies explored it in the context of the COVID-19 pandemic. A study among frontline HCWs working in the acute phase of COVID-19 pandemic in Italy found that higher levels of functioning impairment among individuals with moderate to severe acute post-traumatic stress and depressive symptoms with respect to those without. Acute post-traumatic stress and depressive symptoms were predictive factors of impairment in each domain of functioning analyzed. Another study from Xiao *et al*. revealed that, among 900 HCWs at the peak of the pandemic in China, 55.1% reported psychological distress and 53% had clinically significant depressive symptoms associated with a negative impact on functioning. Data from the present study confirmed the additional toll of comorbidity on psychosocial adjustment: HCWs showing both PTSD and depression reported significantly higher levels of work and social functioning impairment with respect to the others.

When interpreting our results, some limitations should be taken into account. First, the cross-sectional design of the study that does not allow to detect the course of psychopathological symptoms and their impact on HCWs’ functioning in the long-term. Second, the limited sample size, which may have an influence on the generalizability of the results. Third, the use of self-report instruments rather than clinical interviews for the assessment of mental health outcomes and the lack of assessment of other psychopathological symptoms besides post-traumatic stress and depressive ones that may influence the results.

## CONCLUSION

The present study reported on PTSD and depression detected among the first Italian frontline HCWs involved in the management of patients referring to the hospital emergency unit for the symptoms related to the COVID-19 during the initial phase of the COVID-19 outbreak in the country. In this regard, it is important to recall that hospital of Codogno, in Italy was the first European epicenter of the COVID-19 emergency and, to the best of our knowledge, this is the first study investigating mental health outcomes and their interplay in the framework of the COVID-19 epidemic among these HCWs. A high toll in terms of psychological burden emerged, especially for individuals who experienced psychiatric comorbidities, suggesting the absolute need to identify and define adequate programs to support HCWs working in highly stressful circumstances, as those related to the still ongoing COVID-19 health emergency. These data also highlight the need to raise awareness of HCWs to risks for negative psychological outcomes due to their work activity and to the possibility to manage them avoiding chronicity and a global negative impact on different areas of functioning. Further studies in larger samples and with a longitudinal design are warranted, with the aim to better identify HCWs at risk of developing adverse mental health outcomes and to adopt tailored education and training measures for appropriate prevention strategies.

## Figures and Tables

**Fig (1):**
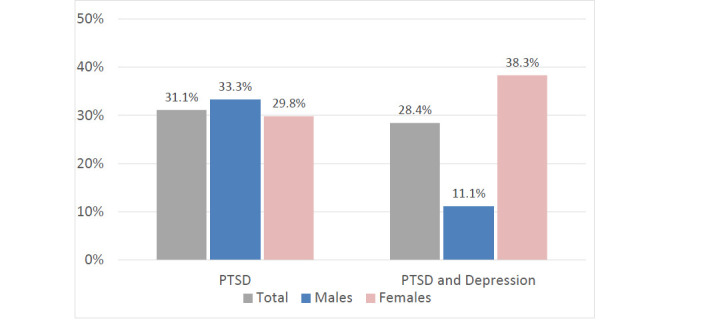
Prevalence rates of PTSD and PTSD and depression in the overall sample (N=74) and divided by gender (Males N=27 and Females N= 47).

**Table 1:** Socio-demographic and work-related characteristics in the overall sample (N=74) and in the No PTSD (N=30), PTSD only (N=23), PTSD and depression (N=21) groups.

**-**	**Variable**	**Total Sample** N(%)	**No PTSD** N (%)	**PTSD Only** N (%)	**PTSD and Depression** N (%)	***p***
**Gender**	Males	27 (36.5)	15 (50.0)	9 (39.1)	3 (14.3)	.032
	Females	47 (63.5)	15 (50.0)	14 (60.9)	18 (85.7)	
**Occupational Role**	Medical doctors	18 (24.3)	9 (30.0)	6 (26.1)	3 (14.3)	.425
	Other HCWs	56 (75.7)	21 (70.0)	17 (73.9)	18 (85.7)	
**Unit**	ER	18 (24.3)	7 (23.3)	8 (34.8)	3 (14.3)	.282
	Other unit	56 (75.7)	23 (76.7)	15 (65.2)	18 (85.7)	
**Management of COVID-19 patients**	Yes	46 (62.2)	18 (60.0)	17 (73.9)	11 (52.4)	.322
	No	28 (37.8)	12 (40.0)	6 (26.1)	10 (47.6)	
**Having assisted to death of COVID-19 patients**	Yes	39 (52.7)	14 (46.7)	15 (65.2)	10 (47.6)	.350
	No	35 (47.3)	16 (53.3)	8 (34.8)	11 (52.4)	
**Increased workload due to COVID-19**	Yes	45 (60.8)	15 (50.0)	14 (60.9)	16 (76.2)	.169
	No	29 (39.2)	15 (50.0)	9 (39.1)	5 (23.8)	
**Having children**	Yes	25 (33.8)	7 (23.3)	10 (43.5)	8 (38.1)	.272
	No	49 (66.2)	23 (76.7)	13 (56.5)	13 (61.9)	
**Relatives got sick with COVID-19**	Yes	35(47.3)	12 (40.0)	12 (52.2)	11 (52.4)	.563
	No	39 (52.7)	18 (60.0)	11 (47.8)	10 (47.6)	
**Death of a relative due to COVID-19**	Yes	13 (17.8)	3 (10.0)	4 (17.4)	6 (28.6)	.230
	No	61 (82.4)	27 (90.0)	19 (82.6)	15 (71.4)	
**Family history of mental disorders**	Yes	15 (20.3)	6 (20)	4 (17.4)	5 (23.8)	.868
	No	59 (79.7)	24 (80)	19 (82.6)	16 (76.2)	
**History of psychiatric treatments**	Yes	14 (18.9)	2 (6.7)	5 (21.7)	7 (33.3)	.052
	No	60 (81.1)	28 (93.3)	18 (78.3)	14 (66.7)	
**Suffering from physical illnesses**	Yes	13 (17.6)	3 (26.1)	6 (26.1)	4 (19.0)	.306
	No	61 (82.4)	27 (10)	17 (73.9)	17 (81.0)	

**Table 2:** Comparisons of socio-demographics and psychometric measures’ scores in the total sample and in the No PTSD, PTSD only, PTSD and depression groups.

	**Total Sample** **(N=74)**	**No PTSD** **(N=30)**	**PTSD Only** **(N=23)**	**PTSD and Depression** **(N=21)**	***p***	**Post-Hoc** **Comparison**
	**Mean±SD**	**Mean±SD**	**Mean±SD**	**Mean±SD**		
Age (Years)	39.3±12.2	38.10±12.4	39.9±11.5	40.6±13.23	.838	-
Hospital duty time (Years)	7.5±10.9	7.4±11.8	8.2±10.5	6.9±10.7	.581	-
Number of family members	2.3±1.4	2.2±1.6	2.3±1.3	2.6±1.2	.539	-
IES-R	38.4±18.6	19.7±7.4	46.0±11.3	56.7±10.5	<.001	a<b**; a<c**
IES-R intrusion	1.9±1.0	0.09±0.05	2.5±0.5	2.9±0.4	<.001	a<b**; a<c**
IES-R avoidance	1.5±7.7	0.9±0.4	1.9±0.7	2.2±0.7	<.001	a<b**; a<c**
IES-R *hyperarousal*	1.5±1.0	0.7±0.3	2.0±0.8	2.6±0.6	<.001	a<b**; a<c**
PHQ-9	7.7±5.2	4.2±2.7	6.3±2.2	14.1±4.2	<.001	a<c**; b<c**
ProQOL Compassion Satisfaction	37.9±6.4	39.0±5.6	38.6±6.4	35.5±7.2	.163	-
ProQOL Burnout	23.4±6.0	20.6±4.0	22.7±5.7	28.1±6.2	<.001	a<c**; b<c**
ProQOL Secondary Traumatic Stress	22.4±8.0	16.7±3.5	22.5±6.0	30.6±7.6	<.001	a<b**; a<c**; b<c*
GAD-7	9.7±5.6	5.4±2.9	9.9±4.7	15.7±3.7	<.001	a<b**; a<c**; b<c**
Resilience Scale	129.0±21.9	134.0±16.0	126.9±26.1	124.4±23.7	.429	-
WSAS	15.7±11.2	9.3±9.6	15.3±8.9	26.1±7.8	<.001	b<c**; a<c**

*p=<.05. **p=<.01. a= No PTSD group. b= PTSD only group. c= PTSD and depression group.

## Data Availability

Data supporting the findings of the article is not publicly available as a result of the privacy policies of the health facilities involved in the study, but it can be provided by the corresponding author [VP], on reasonable request.

## LIST OF ABBREVIATIONS

PTSD: = Post-Traumatic Stress Disorder
HCWs: = Health Care Workers
COVID-19: = Corona Virus Disease-19
